# Statistics of Tænia

**Published:** 1843-07-22

**Authors:** 


### STATISTICS OF TÆNIA.

Herr Wauruch, of Vienna, states that of 3864 persons treated in the space of twenty years at an hospital of that city, 206 were affected with tænia. Of these 206, 71 were males, and 135 females. The oldest individual affected was a man of fifty-four, and the youngest a girl of three years and a half old. Most were persons between fifteen and forty years of age. Tænia was not found in any woman after the cessation of the menses, and in only two men above fifty years years old. Persons most concerned with animal provisions were those observed to be chiefly attacked; for of the 206 patients, 1 was a man, and 52 were women cooks, several were butchers, and 11 were eaters of large quantities of meat. Among predisposing causes, the principal were a habitation in a damp neighbourhood, and the use of injured aliments, as bad bread, flour, potatoes, &c., but particularly bad mutton, pork, and water.—*Ibid, from Osser. Med. Jahrbuch.*

